# Clinical and neuropathological study about the neurotization of the suprascapular nerve in obstetric brachial plexus lesions

**DOI:** 10.1186/1749-7221-4-15

**Published:** 2009-09-11

**Authors:** Dominique Schaakxs, Jörg Bahm, Bernd Sellhaus, Joachim Weis

**Affiliations:** 1 Institute for Neuropathology, Klinikum RWTH Aachen, Germany; 2 Euregio Reconstructive Microsurgery Unit, Franziskushospital Aachen, Germany

## Abstract

**Background:**

The lack of recovery of active external rotation of the shoulder is an important problem in children suffering from brachial plexus lesions involving the suprascapular nerve. The accessory nerve neurotization to the suprascapular nerve is a standard procedure, performed to improve shoulder motion in patients with brachial plexus palsy.

**Methods:**

We operated on 65 patients with obstetric brachial plexus palsy (OBPP), aged 5-35 months (average: 19 months). We assessed the recovery of passive and active external rotation with the arm in abduction and in adduction. We also looked at the influence of the restoration of the muscular balance between the internal and the external rotators on the development of a gleno-humeral joint dysplasia. Intraoperatively, suprascapular nerve samples were taken from 13 patients and were analyzed histologically.

**Results:**

Most patients (71.5%) showed good recovery of the active external rotation in abduction (60°-90°). Better results were obtained for the external rotation with the arm in abduction compared to adduction, and for patients having only undergone the neurotization procedure compared to patients having had complete plexus reconstruction. The neurotization operation has a positive influence on the glenohumeral joint: 7 patients with clinical signs of dysplasia before the reconstructive operation did not show any sign of dysplasia in the postoperative follow-up.

**Conclusion:**

The neurotization procedure helps to recover the active external rotation in the shoulder joint and has a good prevention influence on the dysplasia in our sample. The nerve quality measured using histopathology also seems to have a positive impact on the clinical results.

## Background

Brachial plexus lesions during birth affect one in 2000 newborns [1]. Ten percent of them need early or secondary surgical reconstruction [1]. In the treatment of obstetric brachial plexus lesions, one of the main problems is the poor recovery of abduction and external rotation in the shoulder joint [2].

In children with upper and total brachial plexus lesions, the suprascapular nerve, the first motor branch of the upper trunk located in the center of the obstetric brachial plexus lesion, is usually affected. The clinical manifestation is the lack of active external rotation in the glenohumeral joint. The child adopts an internal rotation position and might be restricted in many activities such as: eating, writing, dressing or combing their hair. Some of them develop a "trumpet sign" posture, indicating that elbow flexion is executed with an abducted arm and a pronated forearm, the supination of the forearm being limited. The lack of external rotation can lead to secondary soft tissue contractures, deformities in the shoulder joint such as a posterior subluxation together with an enhanced retroversion of the humeral head and various glenoid deformities. An important point is to restore the external rotation in order to prevent these deformities [1,3].

The main goal of the plexus reconstruction is the recovery of the motor and sensory functions of the hand, as well as elbow flexion, shoulder stability and motion. Many muscles are involved in the shoulder motion, mainly controlled by four nerves (axillary nerve, deltoid muscle: abduction; suprascapular nerve, supraspinatus and infraspinatus muscles: external rotation of the humerus and abduction in the supraspinatus muscle; dorsal scapular nerve, rhomboidei muscles and long thoracic nerve, seratus anterior muscle: scapular stabilization). The natural balance between the lateral (infraspinatus and supraspinatus muscle) and the medial rotators (latissimus dorsi, teres major, subscapularis, pectoralis major muscles) favors the internal rotation [1].

In our study, we wanted to answer the following questions:

1. Can we get a good recovery of the active external rotation after the spinal accessory nerve neurotization to the suprascapular nerve? What could be the reasons for insufficient results?

2. Does neurotization of the suprascapular nerve reduce the amount of shoulder dysplasia seen by allowing the recovery of muscle balance between the internal and external rotators? Could an existing dysplasia be treated? Is it possible through this procedure to prevent the development of a shoulder dysplasia?

3. Is there a correlation between the quality criteria of the nerves involved in the reconstruction measured by the histopathology (morphometry and microscopic qualitative analysis) and the clinical results? Is it possible to identify clinical prognostic factors with the analysis of these parameters?

## Methods

We examined 65 patients (37 girls and 28 boys) who required brachial plexus reconstruction between 2001 and 2007. We operated on all 65 patients at ages ranging between 5 and 35 months (average: 19 months) and assessed their recovery for a mean postoperative observation period of 2.5 years.

### Surgical techniques

Our 65 patients presented varying grades of severity of obstetric brachial plexus lesions involving the suprascapular nerve. Depending on lesion severity, 3 groups of patients were operated on using different surgical procedures:

1. Accessory nerve neurotization to the suprascapular nerve using the dorsal approach (N = 38). All patients in this group presented an upper brachial plexus palsy.

2. Accessory nerve neurotization to the suprascapular nerve and neurolysis of the other cervical nerve roots: ventral approach (N = 6). All patients in this group presented an upper brachial plexus palsy.

3. Plexus reconstruction on patients with complete brachial plexus lesion and accessory nerve neurotization to the suprascapular nerve: ventral approach (N = 21). Out of these patients, 10 presented a lesion of C5-C7 and 11 had a total brachial plexus palsy.

The dorsal approach has been described previously [1].

For a ventral approach, the patient was placed in a supine position under general anesthesia and orotracheal intubation. A 4 cm horizontal incision was made laterally beginning at the border of the sternocleidomastoideus muscle. The subcutaneous tissue and the platysma were divided and then the adipolymphatic tissue was dissected. The jugularis vein, the carotis and the phrenic nerve were identified. Then, on the scalenus anterior muscle, the phrenic nerve was stimulated. The dissection was carried out far enough proximally (root C4) and distally (under the clavicle) according to the extent of the lesion to expose the brachial plexus. The trunks and the roots of the brachial plexus down to their foramen were progressively identified and individualized by rubber loops [4,5]. In case of neuroma in continuity, a neurolysis can be performed to release the intraneural pressure caused by the scar tissue and favor a good recovery in patients with Erb's palsy [6,7]. Functional recovery was assessed using electrical stimulation. After that, the neurotization of the suprascapular nerve was performed. The suprascapular nerve was followed close to its emergence from the upper trunk and cut. The accessory nerve was followed as distal as possible, cut and the proximal collaterals were spared to protect the horizontal trapezius function. Neuropathology samples were taken and a classic epineural repair by 10-0 sutures or by fibrin glue was performed as distal as possible to reduce the reinnervation time [1]. In case of a simple neurotization, using a dorsal or ventral approach, no cast was needed but only 10 days of immobilization with the elbow against the body.

In the complete plexus reconstruction including an accessory nerve neurotization (ventral approach), the patient was in a supine position, under general anesthesia and orotracheal intubation. The same operative procedure as described above was used to expose the brachial plexus. The topographic anatomy of the different brachial plexus branches was exposed by using electrostimulation and assessing the muscular motor response. When no motor response was obtained, it is a sign that the nerve or its roots are non conducting [7].

In case of severe upper brachial plexus lesions, reconstructive priorities must be defined. The main goal is the recovery of elbow flexion and shoulder stability. In the exploration of the damaged brachial plexus, several plexus reconstruction options are available: the neurolysis, the intraplexal nerve suture (with or without nerve graft) and the nerve transfer. The choice of the reconstruction techniques is individual and depends on the intraoperative findings [7]. In general, better results are obtained with neuroma resection and nerve transplantation than with the neurolysis [6,8]. When the nerve is ruptured, an autologous nerve graft is used. The sural nerve is most often used as donor nerve [7]. The neuroma parts were removed and nerve samples were taken for the neurohistopathology. The sural nerve parts were taken and an interfascicular transplantation was performed. The coaptation is performed under microscope by 10/0 sutures or fibrin glue. Then the accessory nerve neurotization to the suprascapular nerve was performed as described above. The skin was closed, and a handmade well-padded head and neck plaster was used, which was worn for a period of 3 weeks.

### Clinical examination methods

We studied the recovery of the active external rotation and the issue of the shoulder dysplasia.

**Figure 1:**
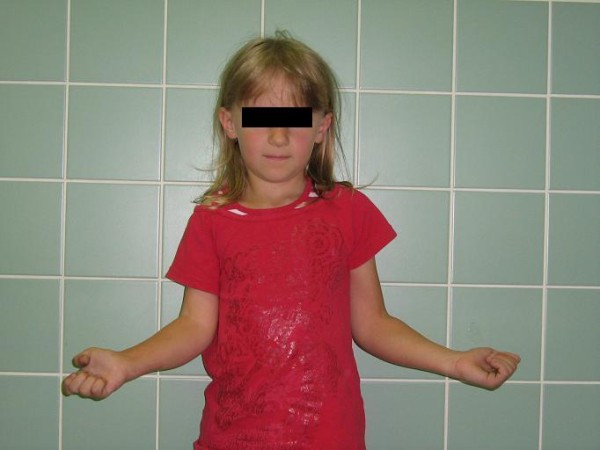
**Active external rotation in adduction**.

**Figure 2:**
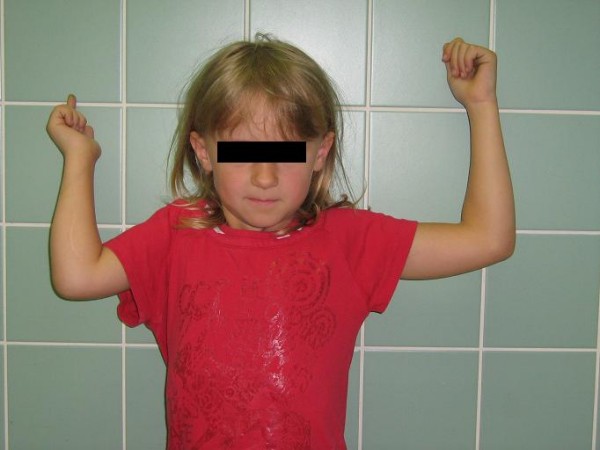
**Active external rotation in abduction**.

We assessed the recovery of external rotation using the range of motion method. Only clinical examination was used, without any device-assisted diagnostic procedure. Different parameters were measured: active and passive external rotation in adduction and in abduction as well as a part of the Mallet score (hand to mouth and hand to head, see Table 1) to assess the functional recovery. For the external rotation in adduction, the neutral position (0°) is with the arm along the lateral chest and the forearm forming a 90° angle with the arm and pointing forwards (see Figure 1). For the external rotation in abduction, the neutral position (0°) is with the arm in 90° abduction and the forearm at a 90° angle with the arm and pointing forwards (see Figure 2).

**Table 1:** Mallet scoring

	0	1	2
Hand to nape of neck	impossible	difficult	Easy

Hand to mouth	impossible	difficult (trumpet sign)	Easy

The glenohumeral joint was also assessed to observe the presence or absence of dysplasia and the impact on the dysplasia of the restoration of the muscle balance (lateral and medial rotators) by means of the neurotization procedure. The mean follow-up period of 2.5 years provides a good indication of the impact of reconstruction on the proper development of the glenohumeral joint, although an additional follow-up after 5 years would be desirable in order to confirm the results. We analyzed the glenohumeral joint clinically without using magnetic resonance imaging (MRI). Although MRI would have been useful from a radiological point of view, it was not possible to carry out this test consistently on a wide sample of young children as it requires general anesthesia, which parents would not have accepted without therapeutical justification. For this reason, we focused on the clinical examination of the joint, checking that there was no major deformity. We assessed the shoulder joint by measuring the range of motion, assessing the presence of contractures and the articular mobility. We stabilized the scapulothoracic joint with one hand and used the other hand to assess the glenohumeral joint external rotation [9]. In case of severe dysplasia, there is an audible "click" during the examination of the passive external rotation and a reduction of the mobility. The literature shows a strong correlation between clinical measures and the presence of dysplasia, detected by MRI [9]. Strongly reduced passive glenohumeral external rotation motion and the presence of internal rotation contracture are indicators of underlying joint deformity.

### Neurohistopathology and morphometry

During the surgical procedures, we took nerve samples of the suprascapular nerve from 13 patients for neurohistopathology. Analyses by microscope and morphometry were carried out at Institute for Neuropathology (Head: Univ. Professor Dr J.Weis), Klinikum RWTH Aachen. Unfortunately, only 11 patients could be compared for clinical and neurohistopathology results due to sample attrition.

### Coloration

Semithin nerve sample sections were obtained by using paraphenylenediamin and toluidine-blue staining to validate the structural details.

### Morphometry

**Figure 3:**
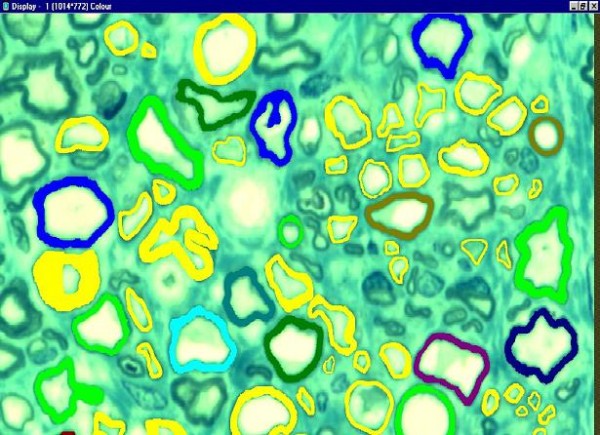
**Morphometry marking process**. Nerve fibers are marked in color. Fibers colored in yellow were selected manually, excluding incomplete fibers located on the edges of the sample or fibers that presented artifacts. Fibers marked in other colors were selected automatically by the KS 300 optical-electronic digital evaluation system.

1 μm semithin sections of the suprascapular nerve from 13 patients were observed under microscope (100×) in oil immersion. We used a KS 300 automatic, optical-electronic digital evaluation system to measure 2 fields per section. All nerve fibers were marked manually, excluding the fibers which were located on the edges of the sample as well as those which were incomplete or had artifacts. For all the fibers that had been marked, various parameters per field were measured: myelin surface (μm^2^), total nerve fiber surface (μm^2^), axon surface (μm^2^), myelin diameter (μm), axon diameter (μm), total nerve fiber diameter (μm) and number of fibers. Figure 3 shows a part of this marking process.

### Quality criteria of the suprascapular nerve

We measured the following parameters from the endoneurium using the morphometry, and calculated different ratios to assess the nerve quality:

- The M/A ratio (surface of myelin (μm^2^)/surface of axon (μm^2^)) gives an indication of the thickness of the myelin in the axon.

- The G-ratio (axon diameter (μm)/total nerve fiber diameter (μm)) is often used in the literature indicating the degree of myelinization of the axon. Normal values are comprised between 0.5 and 0.7 [10].

- The ratio between the surface of the axon (μm^2^) and the total surface of nerve fiber (μm) corresponds to the proportion of axon material in the nerve fascicle. This ratio is comparable to the G-ratio but is more precise, because the shape of the nerve fiber is not exactly round. For this reason, the diameter only gives an approximation of the relative surface in the nerve fascicle.

- The ratio between the surface of myelin (μm^2^) and the total surface of the observed nerve sample (μm^2^) indicates the proportion of myelin in the total observed nerve sample.

- The ratio between the total surface of nerve fiber (μm^2^) and the total surface of the observed nerve sample (μm^2^) indicates the proportion of nerve fiber in the total nerve sample.

Under the microscope, we observed the following qualitative criteria of the suprascapular nerve [5]:

- number and orientation of nerve fascicles

- presence of peri- or endoneural fibrosis

- remnants of nerve degeneration (clusters of Schwann cells called "Büngner" bands)

- indirect signs of reinnervation: presence of minifascicles

- presence or absence of minifascicles in the perineurium or epineurium, which are a sign of neuroma.

**Figure 4:**
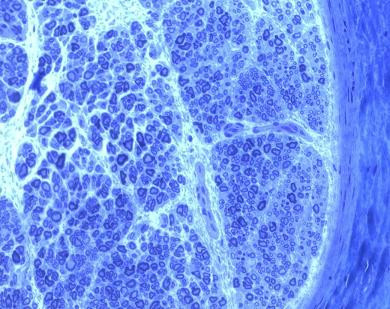
**Suprascapular nerve showing good endoneural regeneration**.

**Figure 5:**
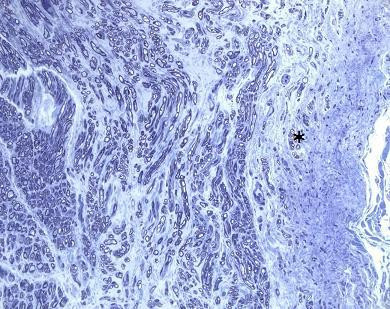
Suprascapular nerve with minifascicles in the perineurium (*).

Figure 4 shows a suprascapular nerve with good endoneural regeneration and Figure 5 shows another suprascapular nerve with minifascicles in the perineurium, which indicates the presence of a neuroma. The higher the presence of minifascicles in the perineurium and epineurium, the more important is the neuroma. Therefore, the presence of minifascicles is an indicator of the lower quality of the nerve involved in the neurorrhaphy, potentially compromising the clinical results.

### Statistical evaluation

The clinical values for the external rotation are measured in degrees, the minimum being 0° (no result) and the maximum 90° (goal value). We assessed the clinical parameters at different times and used a one-sample T-test to show the postoperative improvement of the clinical parameters.

We distinguished between the 3 procedure groups and tried to show the influence of the primary reconstructive procedure on the postoperative results by using a one-way analysis of variance (ANOVA) procedure. Given the relatively small sample and potentially non-normal distribution, we further confirmed this statistic using the non-parametric rank-based Kruskal-Wallis test. In our clinical examinations, we looked also for the presence of dysplasia in the glenohumeral joint (before and after the procedure). We used descriptive statistics and variance analysis to show the influence of the dysplasia on the postoperative results and frequency tables to assess the influence of the neurotization (restoration of the muscle balance) on the dysplasia.

The morphometry criteria were assessed using descriptive statistics.

## Results

### Clinical results

We examined the children (N = 65) postoperatively at different times, which were statistically distributed in different groups:

- 0-6 months after the procedure

- after 7-12 months

- after 13-18 months

- after 19-24 months

- after 25-36 months

- after 36 months or more

Before the operation, all the patients presented an active external rotation (in abduction and in adduction) close to 0°. In general, we observed an improvement of the clinical values in the 3 years following the procedure, as shown in Table 2.

**Table 2:** Clinical result evolution (degrees of rotation achieved)

Average	0-6 months	7-12 months	13-18 months	19-24 months	25-36 months	36+ months	Max. reached
**active external rotation (abduction)**	36.79	47	58.61	64.67	70.37	70.27	67.54

**passive external rotation (abduction)**	68.75	57.92	77.73	79.58	76.43	85.81	86.1

**passive external rotation (adduction)**	77.5	56.67	48.33	47.08	48.93	53.57	56.61

**active external rotation (adduction)**	40	16.25	47.5	25	24.29	28.75	28.1

A one-sample T-test confirmed the improvement of the clinical parameters with all postoperative values being significantly different from the initial value of 0° (p = 0.00).

We calculated the mean of the maximal values reached by all the patients. The maximum goal value for the different clinical parameters is 90° of rotation. For the active external rotation in abduction parameter, 71.5% of patients reached a value comprised between 60°-90°. For passive external rotation in abduction, 96.6% of patients reached 70°-90°. For passive external rotation in adduction, 50% of patients obtained a value between 60° and 90°. The results for active external rotation in adduction were more disappointing with only 18% of patients obtaining 60°-90°, while 66% reported 0°-30°. The detailed frequencies are shown in Table 3.

**Table 3:** Clinical result frequencies (maximum degrees of rotation achieved)

Maximum degrees achieved (Percent of patients)	Active external rotation (Abduction)	Passive external rotation (Abduction)	Active external rotation (Adduction)	Passive external rotation (Adduction)
**0-10**	6.3	0.0	36.0	3.6

**11-20**	3.2	0.0	8.0	3.6

**21-30**	6.3	0.0	22.0	16.1

**31-40**	4.8	1.7	8.0	7.1

**41-50**	6.3	1.7	8.0	17.9

**51-60**	6.3	0.0	8.0	10.7

**61-70**	12.7	6.8	0.0	12.5

**71-80**	9.5	6.8	6.0	10.7

**81-90**	44.4	83.1	4.0	17.9

In general, we observe better results for the external rotation in abduction than in adduction. Active external rotation improves gradually up until 3 years following the procedure, then stabilizes. Passive external rotation decreases at first, maybe due to the immobilization following the procedure. Evolutions from one period to the next should be considered with some caution as the patients observed are not always the same for practical reasons. We observe better results for passive than for active values (see Table 4).

**Table 4:** Comparison of maximum results achieved between exercise types

Patients achieving 90° rotation after 36 months	Abduction	Adduction
**Active**	37.8%	3.1%

**Passive**	81.1%	11.4%

We sought an explanation for the unsatisfactory results achieved by some patients in their individual follow-up (active external rotation in abduction between 0° and 20°). Two patients could not obtain any active external rotation in abduction. One of them had no active external rotation in abduction 5.5 months after the procedure and did not show up for the next examination. The other one, who underwent a ventral approach with a neurolysis from the trunci superior and medius and a neurotization of the suprascapular nerve developed a shoulder contracture.

Three patients reached an active external rotation in abduction comprised only between 0°-20° because they developed a shoulder contracture. Another patient with a heavy subtotal plexus lesion reached an active external rotation in abduction of 10° eight months after the operation and did not come to the examination afterwards.

The Mallet score results also show marked improvement over the initial values. For hand-to-mouth, the value reached was an average of 1.90, with 10.2% of patients reaching the value of 1 and 89.8% reaching the maximum value of 2. For hand-to-head movement, 84.7% of patients registered an improvement with an average maximum value of 1.39.

We also examined the influence of the type of the primary reconstructive operation on the clinical results. We used a one-way ANOVA procedure as well as a Kruskal-Wallis test to test for differences in the clinical results in our 3 operation groups. We obtained significant differences between the 3 operation groups for active external rotation in abduction (ANOVA p = 0.026, Kruskal-Wallis p = 0.036), passive external rotation in abduction (ANOVA p = 0.017, Kruskal-Wallis p = 0.048) and Mallet Score parameters (ANOVA p = 0.000, Kruskal-Wallis p = 0.000 for both Hand-to-Mouth and Hand-to-Head). The patients who underwent only the neurotization operation obtained better results than the patients who had complete plexus reconstruction. The descriptive statistics for the maximum values reached for each clinical parameter in each operation group are shown in Table 5. We did not find any significant difference between the 3 groups for parameters passive external rotation in adduction (ANOVA p = 0.198, Kruskal-Wallis p = 0.471) and active external rotation in adduction (ANOVA p = 0.447, Kruskal-Wallis p = 0.568). It should be cautioned that this result is in need of verification as one of the three groups (patients having undergone neurotization and neurolysis) was much smaller (N = 6) than the other groups (N = 38 and 21, respectively).

**Table 5:** Descriptive statistics for clinical parameters of different operation groups (ANOVA analysis)

		N	Mean	Std. Deviation	95% Confidence Interval for Mean		Min	Max
					**Low**	**High**		

**Active external rotation (abduction) - max. reached**	Neurotisation N. XI/SSC	38	74.74	22.480	67.35	82.13	0	90
	Neurotisation+ Neurolysis	6	61.67	33.116	26.91	96.42	0	90
	Complete Plexus reconstruction	19	55.00	29.627	40.72	69.28	10	90
	Total	63	67.54	26.984	60.74	74.34	0	90
								
**Passive external rotation (abduction) -- max. reached**	Neurotisation N. XI/SSC	35	88.71	3.900	87.37	90.05	70	90
	Neurotisation+ Neurolysis	6	77.50	17.819	58.80	96.20	45	90
	Complete Plexus reconstruction	18	83.89	12.897	77.48	90.30	40	90
	Total	59	86.10	9.916	83.52	88.69	40	90
								
**Passive external rotation (adduction) -- max. reached**	Neurotisation N. XI/SSC	33	59.70	21.612	52.03	67.36	5	90
	Neurotisation+ Neurolysis	6	40.83	28.358	11.07	70.59	10	80
	Complete Plexus reconstruction	17	56.18	24.656	43.50	68.85	20	90
	Total	56	56.61	23.551	50.30	62.91	5	90
								
**Active external rotation (adduction) -- max. reached**	Neurotisation N. XI/SSC	30	31.17	25.281	21.73	40.61	0	90
	Neurotisation+ Neurolysis	4	13.75	24.281	-24.89	52.39	0	50
	Complete Plexus reconstruction	16	25.94	30.068	9.92	41.96	0	90
	Total	50	28.10	26.743	20.50	35.70	0	90
								
**Hand-Head -- max. reached**	Neurotisation N. XI/SSC	36	1.67	.586	1.47	1.86	0	2
	Neurotisation+ Neurolysis	6	.50	.548	-.07	1.07	0	1
	Complete Plexus reconstruction	17	1.12	.781	.72	1.52	0	2
	Total	59	1.39	.743	1.20	1.58	0	2
								
**Hand-Mouth - max. reached**	Neurotisation N. XI/SSC	36	2.00	.000	2.00	2.00	2	2
	Neurotisation+ Neurolysis	6	1.50	.548	.93	2.07	1	2
	Complete Plexus reconstruction	17	1.82	.393	1.62	2.03	1	2
	Total	59	1.90	.305	1.82	1.98	1	2

In our clinical examinations, we also looked for the presence of dysplasia in the glenohumeral joint (pre and post-operatively). The patients showed a muscular imbalance between the external rotators and the internal rotators in favor of the internal rotators. The neurotization operation contributes to the restoration of the muscular balance in the glenohumeral joint and should have a positive influence on the shoulder joint and therefore prevent the development of a shoulder dysplasia. In our sample, 7 patients affected by dysplasia before the operation did not show any sign of dysplasia in the postoperative follow-up. Although this is a small sample, we observe a positive influence from the reconstructive operation on the glenohumeral joint.

### Histopathology results

We looked for tendencies in the relation between the histopathology and clinical results. The histopathology results in our 13 samples were normal on average, as shown in Table 6. In particular, the G Ratio (axon diameter (μm)/total nerve fiber diameter (μm)) is often used in the literature to indicate the degree of myelinization of the axon. Normal values are comprised between 0.5 and 0.7 [10]. In our results, all the G Ratio values were contained within this interval, with the exception of one value which was very close to the normal range (0.48) and one patient with a G Ratio value of 1.

**Table 6:** Histopathological parameters of nerve samples

	Mean	**Std. Dev**.
**G Ratio**	0.59	0.07

**Myelin surface/Axon surface**	4.53	4.59

**Axon surface/Total nerve surface **	0.39	0.09

**Myelin proportion in total sample**	0.15	0.04

**Nerve fiber proportion in total sample**	0.25	0.08

We also assessed the number and orientation of the nerve fascicles, the presence of perineural or endoneural fibrosis, signs of regeneration and the presence of minifascicles in the perineurium or in the epineurium to check if these criteria could influence the clinical results. Most patients did not have minifascicles in the perineurium or in the epineurium and showed signs of good endoneural regeneration and no sign of degeneration. Two of our patients showed a very small presence of minifascicles in the perineurium with signs of endoneural regeneration. Both patients achieved good clinical results. One patient had a very small presence of minifascicles in the perineurium and in the epineurium, good endoneural regeneration and achieved good clinical results as well. Only one patient, who presented an important lesion treated by complete plexus reconstruction and the neurotization of the suprascapular nerve showed an important neuroma (high number of minifascicles in the perineurium and in the epineurium) and a G Ratio value of 1. The clinical results for this patient were insufficient in the follow-up, leading us to suspect a problem of quality of the suprascapular nerve involved in the neurorrhaphy with the accessory nerve.

## Discussion

The lack of recovery of active external rotation is an important problem in children suffering from brachial plexus lesions involving the suprascapular nerve. The restoration of the rotational balance between the internal and external rotators is important to the good development of the shoulder motion and to prevent deformities of the glenohumeral joint in patients suffering from brachial plexus palsy.

The standard procedure is the transfer of the distal branch of the accessory nerve to the suprascapular nerve. The indication for this procedure is the lack of active lateral rotation in the glenohumeral joint without restriction of passive external rotation (i.e. only internal rotation position, but no joint contracture) for children younger than 2 years [1,11]. It has been shown in the literature that this procedure provides an improvement in active external rotation of the shoulder [1,2,12-17].

Our study contributes to the understanding of this problem in the following ways. Firstly, we confirm earlier results and add finer detail by differentiating between operative groups of differing lesion severity. We also examined the cases for which no satisfactory results were obtained. Secondly, we study the impact of the neurotization procedure on the shoulder dysplasia. Lastly, we examine the relation between the histopathology of the nerve samples and the clinical results. In the literature, most surgeons report better clinical results by using the accessory nerve as donor, which provides enough motor power, instead of grafts from the ruptured C5 root [5,18] for the neurotization of the suprascapular nerve. Others did not find any significant difference for the restoration of true external rotation between nerve grafting from C5 and extraplexal nerve transfer using the accessory nerve, but observed a slightly smaller passive range of motion and a slightly stronger tendency to develop an internal rotation contracture in the C5 graft group. In those cases, the recovery of fair range of glenohumeral external rotation was disappointingly low. However, these compensatory techniques seem to contribute to reach a considerable range of movement [16]. Other surgeons performed a reconstruction of the suprascapular nerve by using a direct neurotization with the accessory nerve or by using an interposition nerve graft and obtained better results with the direct neurotization [17].

In our sample, most patients obtained good recovery of the external rotation in abduction. A possible explanation for patients presenting good intraoperative conductivity of the suprascapular nerve and good muscular response but an insufficient recovery of the external rotation could lie in insufficient cortical integration or some co-contraction patterns [1].

Better results were obtained for the external rotation in abduction than for the external rotation in adduction. A possible explanation for this finding is that in adduction, strong internal rotator muscles (the subscapularis muscle, the teres major, the latissimus dorsi and the pectoralis major) work against this movement. In abduction, external rotators (the strong infraspinatus, the teres minor, and the posterior fibers of the deltoid) support the movement. The main internal rotator (the subscapularis muscle) does not act counter to the passive external rotation because it is defunctioned as an internal rotator of the shoulder when the arm is abducted to 90°. This is due to the tendon of the subscapularis being co-axial with the humerus in that position and therefore unable to provide a force vector producing internal rotation. We also observed better results in the passive movement than in the active movement and a positive correlation between both parameters. The passive motion recovery is important to obtain satisfactory active motion recovery because the glenohumeral joint has to be free in order to allow the improvement of the external rotation motion. In order to free a blocked shoulder joint, other operative procedures are used, such as an anterior release with coracoid shortening osteotomy and subscapular tendon lengthening [1].

In our 3 patient groups, we obtained better results for the external rotation in abduction for the patients who only underwent the neurotization operation than for patients who had complete reconstruction, including the neurotization of the suprascapular nerve. We would expect to see more restriction for the external rotation in the group affected by Erb's palsy than in the group affected by total palsy, because the medial rotators are equally affected in the complete plexus lesion, and in this case there is never an imbalance or a rotational contracture of the shoulder [3]. A possible cause for the lack of external rotation recovery in patients with complete palsy could be that the supraspinatus muscle involving in the external rotation movement, being the first to be reinnervated, attracts more axons than the infraspinatus. Another possible cause could be an unsatisfactory cortical integration of this movement or the presence of some pathologic co-contraction pattern. [1,14]

In our clinical evaluation, we also looked at the impact of the neurotization procedure on the dysplasia. In our sample, neurotization of the suprascapular nerve reduces the amount of shoulder dysplasia observed by allowing the recovery of muscle balance between the internal and external rotators. Seven patients showing a dysplasia before the neurotization operation no longer showed signs of dysplasia in the postoperative follow-up. Furthermore, we observed that this procedure can prevent the development of a dysplasia of the glenohumeral joint, as no patient in our sample developed dysplasia postoperatively.

These results point to a positive relation between the histopathological quality of the nerves and the clinical results of the procedure. This finding is naturally in need of further empirical confirmation due to the small size of the sample. However, these results are similar to previous observations reported in the literature [15].

## Conclusion

In conclusion, we believe the accessory nerve neurotization to the suprascapular nerve is a safe and reliable procedure, which provides a good recovery of the active external rotation and a positive influence on the shoulder dysplasia development. Different points are important for the success of the operation: (1) the evaluation of the lesion, which is assessed by using electrostimulation during the operative procedure, (2) the anastomosis between the two nerves (so nerve quality needs to be assessed), (3) the problem of the atrophy of the muscles (so the procedure has to be carried out at an early age), (4) the development of co-contraction patterns and shoulder dysplasia and (5) training the movements in order to stimulate the target muscles.

## Abbreviations

N: nerve; M(m): muscle(s); μm: micrometer.

## Consent

Written informed consent was obtained from the patient for publication of this case report and accompanying images. A copy of the written consent is available for review by the Editor-in-Chief of this journal.

## Competing interests

The authors declare that they have no competing interests.

## Authors' contributions

DS participated in the design of the study, assisted on surgical procedures, carried out clinical examinations and morphometry measurements, did the statistical analysis and drafted the manuscript. JB conceived of the study, participated in its design and coordination, carried out the surgery on the patients and the clinical examinations and reviewed the manuscript. JB also obtained the informed consent from the patients for participation in, and publication of, this study, including accompanying photographs, and is available to provide any additional information in this regard to the Editor-in-Chief of this journal. BS and JW participated in the design and coordination of the study and carried out the histopathology analysis. All authors read and approved the final manuscript.
